# Dr. Julius J. Boyle

**Published:** 1905-07

**Authors:** 


Dr. Julius J. Boyle, a graduate of the University of Buffalo, 1869, died at his home in Susquehanna, Pa., May 12, 1905, aged 58 years.

